# Vitamin A and E Nutritional Status in Relation to Leptin, Adiponectin, IGF-I and IGF-II in Early Life - a Birth Cohort Study

**DOI:** 10.1038/s41598-017-18531-3

**Published:** 2018-01-08

**Authors:** Qinwen Du, Zhong-Cheng Luo, Anne Monique Nuyt, Francois Audibert, Pierre Julien, Shu-Qin Wei, Dan-Li Zhang, William Fraser, Emile Levy

**Affiliations:** 1 Ministry of Education-Shanghai Key Laboratory of Children’s Environmental Health, Xinhua Hospital, Shanghai Jiao-Tong University School of Medicine, Shanghai, 464200 China; 2 Department of Obstetrics and Gynecology, Sainte-Justine Hospital Research Center, University of Montreal, Montreal, H3T 1C5 Canada; 3 Department of Pediatrics, University of Montreal, Montreal, H3T 1C5 Canada; 4 Departments of Medicine and Endocrinology and Nephrology, CHU-Quebec Laval University Research Center, Quebec City, G1V 4G2 Canada; 5 Department of Obstetrics and Gynecology, University of Sherbrooke, Sherbrooke, J1H 5N4 Canada; 6 Department of Nutrition, Sainte-Justine Hospital Research Center, University of Montreal, Montreal, H3T 1C5 Canada

## Abstract

The metabolic health effects of vitamin A and E nutritional status in early life are largely unknown. We assessed whether vitamin A and vitamin E nutritional status may affect circulating leptin, adiponectin, insulin-like growth factor (IGF)-I and IGF-II levels in early life in humans. In a singleton birth cohort (n = 248), vitamin A and E nutritional status in fetuses/newborns were assessed by cord plasma concentrations of retinol, β-carotene, α- and γ-tocopherols. The primary outcomes were cord plasma leptin, adiponectin, IGF-I and IGF-II concentrations. Cord plasma retinol was significantly positively correlated to IGF-I in girls (r = 0.42, *P* < 0.0001) but not in boys (r = 0.14, *P* = 0.11). Adjusting for maternal and newborn’s characteristics, one log unit increase in cord plasma retinol was associated with a 28.0% (95% CI: 11.1–47.5%) increase in IGF-I in girls (*P* < 0.001) but not in boys (*P* = 0.75). One log unit increment in cord plasma α-tocopherol was associated with a 6.6% (0.4–12.3%) decrease in adiponectin (*P* = 0.04), while one log unit increment in cord plasma γ-tocopherol was associated with a 21.2% (4.7–34.8%) decrease in leptin (*P* = 0.01). There may be a sex-specific association between retinol and IGF-I, a negative association between α-tocopherol and adiponectin, and a negative association between γ-tocopherol and leptin in early life in humans.

## Introduction

Vitamins A and E are essential micronutrients for health^1,2^. However, the metabolic health effects of vitamin A and E nutritional status in early life remain largely unknown. The common circulating forms of vitamins A and E include α- and γ-tocopherols for vitamin E^3^, retinol for vitamin A, and β-carotene for provitamin A^4^. These fat-soluble compounds have been implicated in the normal function of multiple physiological systems^5,6^. The major circulating form of vitamin E is α-tocopherol, whereas γ-tocopherol is the most abundant form of vitamin E in the diet in North America^7^. Surprisingly, high-dose vitamin E (α-tocopherol) supplementation has been associated with increased risk of low birth weight^8^– a condition linked to insulin-like growth factor I (IGF-I) insufficiency^9^. A positive correlation has been observed between serum β-carotene and IGF-I concentrations in adults^10^. Circulating vitamin A and E concentrations have been inversely correlated to adiposity and insulin sensitivity in children^11^. Vitamins A and E may be beneficial for metabolic health through their anti-oxidant effect^12^. An *in vivo* study reported that α-tocopherol supplementation decreased oxidative stress and improved insulin sensitivity in diet induced obese mice^13^. A recent study reported that carotenoids might help prevent the development of type 2 diabetes^14^. Taken together, these observations suggest that vitamins A and E may affect metabolic health. However, there is a lack of data on whether vitamin A and E nutritional status may be associated with metabolic health biomarkers in early life in humans. To fill this important research data gap, we sought to evaluate whether vitamin A and E nutritional status in neonates are associated with circulating concentrations of leptin, adiponectin, IGF-I and IGF-II which are important biomarkers of fetal growth and metabolic health^15,16^. We hypothesized that vitamin A and E nutritional status may affect fetal circulating leptin, adiponectin, IGF-I and IGF-II levels.

## Methods

### Study design

This study was based on a birth cohort described previously^17^. Briefly, a total of 339 singleton pregnant women at 24–28 weeks of gestation were recruited from three obstetric care centers in Montreal between August 2006 and December 2008. Women with major preexisting illnesses (such as chronic diabetes mellitus) were not eligible. Data and specimens were collected at 24–28 and 32–35 weeks of gestation and delivery. In the original birth cohort, we had collected cord blood specimens in 255 infants, but 7 without sufficient plasma specimen (0.5 ml) available for assays of vitamins A and E. The present study included all 248 subjects with cord plasma specimens available for assays of vitamins A and E. All newborns are free of birth defects, infections and respiratory distress syndrome.

### Ethics statement

The study was approved by the Research Ethics Committee of Sainte-Justine Hospital Research Center, University of Montreal, and adhered to the tenets and guidelines of the Declaration of Helsinki.

Written informed consent was obtained from all participants.

### Specimens and assays

Plasma specimens were stored in multiple aliquots in a −80 °C freezer until assays. All assays were completed at 12–18 months after the specimen collection. There were no significant correlations (all *P* > 0.1) between specimen storage time and measurement values for all assays.

For assessing vitamin A and E nutritional status in fetuses/neonates, retinol, β-carotene, α-tocopherol and γ-tocopherol in EDTA cord plasma specimens were measured by high-performance liquid chromatography (HPLC)^18^. Briefly, plasma samples were thawed in the dark and processed for analysis under subdued light. Aliquots (500 μL) of the specimens were mixed for 30 seconds on a vortex mixer with 500 μL of an internal standard (12 μg tocopherol acetate in anhydrous alcohol). After the addition of n-hexane (twice, 2.5 mL each), the tubes were shaken (10 min), sonicated (3 min), and centrifuged (5 min) at 1000 × g at 4 °C. The n-hexane layer was transferred to a tube and the pooled organic extracts of each sample were evaporated to dryness under a gentle stream of nitrogen at 20 °C. After rapidly removing the tubes from the water bath, the residues were reconstituted with 150 μL acetonitrile: methylene chloride: methanol (70:20:10, by volume). After the addition of 25 μg ascorbic acid in 50 μL ethanol, the tubes were mixed in a vortex mixer (30 s) and sonicated (3 min). Aliquots (20 μL) were injected into the HPLC system. The chromatographic analyses were performed on a model 1090 HPLC system (Hewlett-Packard, Montreal) with a spherical 5-μm C18 octadesilsilane hypersil column (20 cm × 2.1 mm internal diameter; Hewlett-Packard). All manipulations were carried out under subdued light to avoid photoisomerization of the compounds. Using this Method, we could determine plasma concentrations (μmol/L) of retinol, β-carotene, α-tocopherol and γ-tocopherol simultaneously in a single experiment. The intra- and inter-assay coefficients of variation (CVs) were in the range of 3.2% to 7.9%.

The assay methods for IGF-I, IGF-II, leptin and adiponectin have been reported previously^15,19^. Total IGF-I was measured by an automated solid-phase, enzyme-labeled chemiluminescent assay, total plasma IGF-II by a human IGF-II ELISA kit (Mediagnost, Aspenhaustr, Germany), leptin by a human leptin immunoassay kit (SPI-Bio, Montigny le Bretonneux, France), and total adiponectin by a human adiponectin immunoassay kit (Invitrogen, Camarilo, USA). The intra- and inter-assay CVs were in the range of 2.0% to 10.4%.

### Co-variables

The available study co-variables included maternal gestational diabetes (yes/no), age (>35 y: yes/no), ethnicity (White, others), parity (primiparous: yes/no), education (university: yes/no), pre-pregnancy body mass index (BMI; kg/m^2^), smoking (yes/no), alcohol use (yes/no), mode of delivery (vaginal, C-section), infant sex (boy/girl), gestational age (weeks) and birth weight (z score). Gestational diabetes was defined according to the 2003 American Diabetes Association’s 2-hour 75 g oral glucose tolerance test (OGTT) diagnostic criteria (the then-current clinical diagnostic criteria): gestational diabetes was diagnosed if the woman had two of three glucose values exceeding the following cutoffs: fasting 5.3 mmol/l, 1-h 10.0 mmol/l, and 2-h 8.6 mmol/l^20^. Birth weight z score was calculated based on the Canadian sex- and gestational age-specific birth weight standards^21^.

### Outcomes

The main outcomes were cord plasma IGF-I, IGF-II, leptin and adiponectin concentrations.

### Statistical analysis

Descriptive statistics on vitamin data are expressed as median, mean ± SD. Data on biomarkers (IGF-I, IGF-II, leptin and adiponectin) and vitamins (retinol, β-carotene, α-tocopherol and γ-tocopherol) were log-transformed in correlation and regression analyses. Partial correlation was used to assess the relationships between vitamins and biomarkers adjusting for gestational age at blood sampling and delivery. Generalized linear models were used to assess the adjusted changes in cord plasma leptin, adiponectin, IGF-I and IGF-II concentrations in relation to per log unit increase in each vitamin measurement controlling for co-variables/potential confounders (maternal and newborn’s characteristics). The sample size (n = 248) in multivariate models is sufficient since linear regression models require only a minimal of two subjects per variable for adequate estimation of regression coefficients, standard errors, and confidence intervals^22^. Interactions between variables were examined in generalized linear models. In the presence of significant interaction (p < 0.05), the associations in separate strata (e.g. boys, girls) were presented. The numbers of missing data were low (0–2%) for all study variables, and observations with missing data were allowed to drop-out in multivariate regression analyses.

SAS (version 9.2; SAS Institute, Cary, NC) was used to perform statistical analyses. For correlation coefficients, *P* values < 0.0025 were considered statistically significant if accounting for multiple tests: 5 primary exposures (retinol, β-carotene, α-tocopherol, γ-tocopherol and γ-:α-tocopherol ratio) in relation to 4 primary outcomes (leptin, adiponectin, IGF-I and IGF-II), the total number of primary correlations: 5*4 = 20, Bonferroni adjusted *P* value cutoff = 0.05/20 = 0.0025. For the adjusted association analyses in generalized linear models controlling for multiple co-variables, *P* values < 0.05 were considered significant.

With alpha error at 5%, the study (n = 248) had a power of >90% to detect an absolute correlation coefficient of 0.2 or greater in the associations of vitamin measurements with cord plasma leptin, adiponectin, IGF-I and IGF-II accounting for multiple tests.

## Results

The characteristics of the study birth cohort were described previously^17^. Most study subjects were White (69%). There were 11 preterm deliveries (4.4%, all moderate and late preterm births, 32–36 weeks), 26 pregnancies complicated by gestational diabetes (10.5%), and 131 boys (52.8%).

Table 1 presents the descriptive statistics for cord plasma concentrations of retinol, β-carotene, α- and γ-tocopherols. The median concentrations were 1.00 μmol/L for retinol, 0.038 μmol/L for β-carotene, 5.71 μmol/L for α-tocopherol and 0.75 μmol/L for γ-tocopherol, respectively. There were no differences in cord plasma concentrations of retinol, β-carotene, α- and γ-tocopherols between pregnancies with vs. without gestational diabetes, and between newborns of preterm vs. term deliveries (all *P* > 0.1; data not shown). The descriptive statistics for cord plasma leptin, adiponectin, IGF-I and IGF-II concentrations in the birth cohort were described previously^15,17,19^. The median concentrations were 25.2 ng/ml for leptin, 19.8 μg/ml for adiponectin, 7.6 nmol/L for IGF-I and 66.4 nmol/L for IGF-II, respectively.

**Table 1:** Cord plasma concentrations of retinol, β-carotene, α-and γ-tocopherols in the study birth cohort (n = 248).

	Median	Mean ± SD
Retinol (μmol/L)	1.00,	1.04 ± 0.49
β-Carotene (μmol/L)	0.038	0.038 ± 0.039
α-Tocopherol (μmol/L)	5.71	5.22 ± 3.05
γ-Tocopherol (μmol/L)	0.75	0.80 ± 0.46
γ-:α-Tocopherol ratio	0.14	0.23 ± 0.24

**Figure 1:**
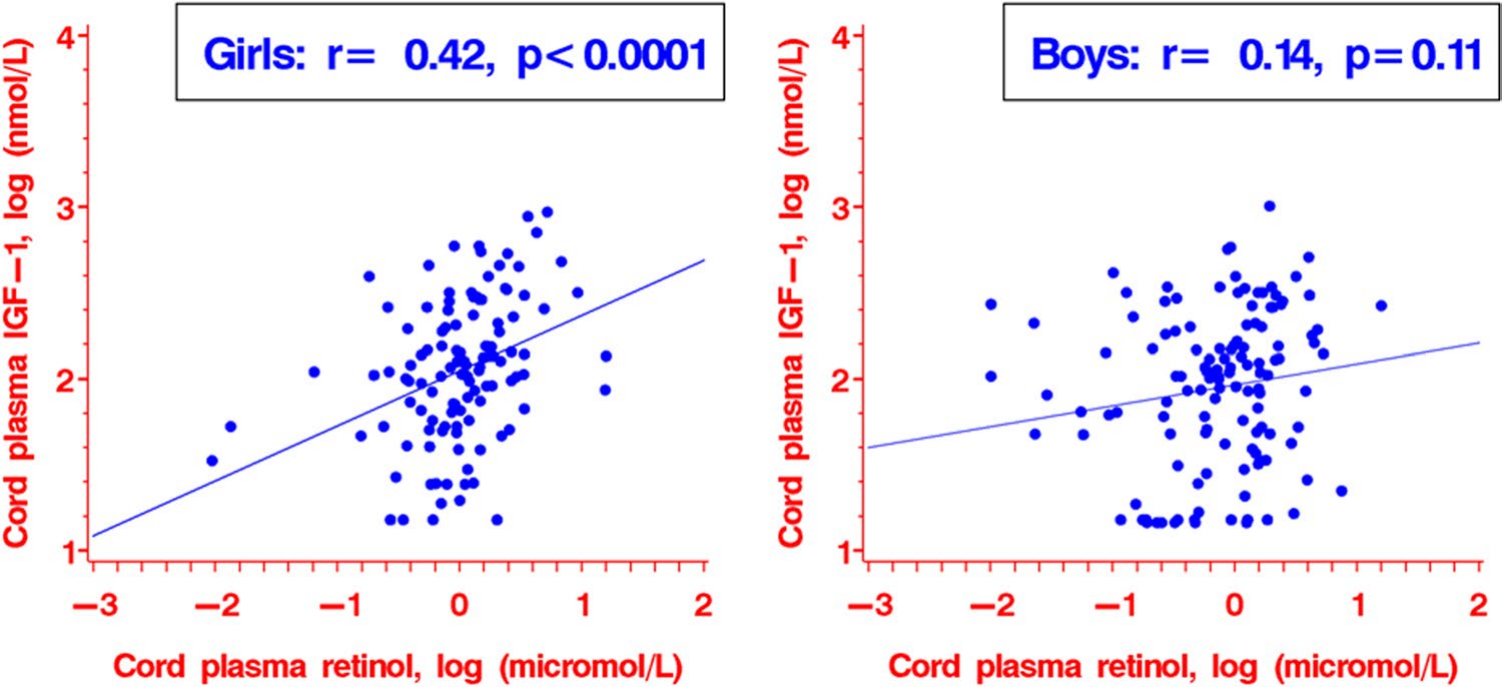
Scatterplots illustrating the differential correlations between cord plasma retinol and IGF-I concentrations in girls (r = 0.42, *P* < 0.0001) and boys (r = 0.11, *P* = 0.14).

Table 2 presents the partial correlations of cord plasma retinol, β-carotene, α- and γ-tocopherols with leptin, adiponectin, IGF-I and IGF-II in newborns adjusting for gestational age. Cord plasma α-tocopherol concentrations were negatively correlated with adiponectin concentrations (r = −0.13, p = 0.049), while γ-tocopherol concentrations were negatively correlated with leptin concentrations (r = −0.15, *P* = 0.02). Retinol concentrations were positively correlated with IGF-I concentrations (r = 0.25, *P* < 0.001) and birth weight (z score) (r = 0.16, *P* < 0.05) overall. Stratified analyses showed that the correlation between cord plasma retinol and IGF-I was only significant in girls (r = 0.42, *P* < 0.0001), but not in boys (r = 0.14, *P* = 0.11) (Fig. 1). There was no evidence of sex-specific associations in other correlations (tests for interactions, all *P* > 0.1).

**Table 2:** Correlations^a^ of cord plasma retinol and β-carotene, α-and γ-tocopherols with leptin, adiponectin, IGF-I and IGF-II in newborns (n = 248).

r^*a*^	Retinol	β-Carotene	α-Tocopherol	γ-Tocopherol	γ-:α- Tocopherol ratio
Leptin	0.11	−0.07	0.01	**−0**.**15** ^**c**^	**−0**.**13** ^**c**^
Adiponectin	0.02	0.05	**−0**.**13** ^**c**^	−0.08	0.02
IGF-I	**0**.**25** ^**b**,**e**^	−0.07	−0.10	−0.01	0.07
IGF-II	0.05	−0.09	−0.02	0.03	0.04
Birth weight	**0**.**16** ^**c**^	−0.11	−0.07	0.01	0.06

^a^Data presented are partial correlation coefficients between vitamins and biomarkers in log-transformed data adjusting for gestational age at blood sampling.

^b^There were differential correlations between cord plasma retinol and IGF-I by infant sex: r = 0.42 (p < 0.0001) for girls, and r = 0.14 (p = 0.11) for boys (*P* = 0.003 for interaction). There was no evidence of differential correlations by infant sex or ethnicity (White, others) for all other correlations.

^c^ *P* < 0.05; ^d^ *P* < 0.0025; ^e^ *P* < 0.001; if accounting for multiple tests, *P* < 0.0025 were considered statistically significant.

Table 3 presents the adjusted associations of cord plasma retinol, β-carotene, α- and γ-tocopherols with leptin, adiponectin, IGF-I and IGF-II in newborns. Adjusting for maternal and newborn’s characteristics, one log unit increase in cord plasma α-tocopherol was associated with a 6.6% (0.4–12.3%) decrease in adiponectin (*P* = 0.04), while one log unit increase in cord plasma **γ**-tocopherol was associated with a 21.2% (4.7–34.8%) decrease in leptin (*P* = 0.01). One log unit increase in γ-:α-tocopherol ratio was associated with a 15.6% (1.0–28.0%) decrease in leptin (*P* = 0.04). One log unit increase in cord plasma retinol was associated with a 9.3% (0.6–18.7%) increase in IGF-I (*P* = 0.04), and a 0.28 (0.08–0.48) increase in birth weight z score (*P* = 0.007) in the whole birth cohort. Cord plasma IGF-II was not associated with any vitamin measurement (retinol, β-carotene, α- or γ-tocopherol).

**Table 3:** Adjusted changes in cord plasma leptin, adiponectin, IGF-I and IGF-II in relation to per log unit increase in retinol, β-carotene, α- and γ-tocopherols in newborns (n = 248).

% change (95% CI)^a^	Retinol	β-Carotene	α-Tocopherol	γ-Tocopherol	γ:α-Tocopherol ratio
Leptin	12.1 (−13.5, 45.2)	−1.1 (−16.1, 16.6)	−0.5 (−19.1, 22.4)	**−21**.**2 (−34**.**8**, **−4**.**7)** ^*^	**−15**.**6 (−28**.**0**, **−1**.**0)** ^*^
Adiponectin	0.9 (**−**6.9, 9.5)	3.8 (**−**1.4, 9.2)	**−6**.**6 (−12**.**3**, **−0**.**4)** ^*^	**−**3.7 (**−**9.3, 2.2)	1.4 (**−**3.5, 6.7)
IGF-I	**9**.**3 (0**.**6**, **18**.**7)** ^*,**b**^	**−**1.5 (**−**6.6, 4.0)	0.4 (**−**6.0, 7.3)	**−**2.5 (**−**8.5, 3.8)	**−**2.3 (**−**7.3, 3.0)
IGF-II	1.6 (**−**3.5, 7.0)	**−**2.5 (**−**5.7, 0.7)	0.4 (**−**3.6, 4.6)	1.4 (**−**2.5, 5.4)	0.8 (**−**2.4, 4.2)
Birth weight (z score)	**0**.**28 (0**.**07**, **0**.**48)** ^*^	**−**0.09 (**−**0.22, 0.04)	**−**0.03 (**−**0.20, 0.13)	**−**0.002 (**−**0.16, 0.15)	0.018 (**−**0.11, 0.14)

^a^Data presented are the percentage change (95% CI) in each biomarker outcome (leptin, adiponectin, IGF-I, IGF-II) per log unit increase in each vitamin measurement, adjusted for gestational diabetes, pre-pregnancy BMI, maternal ethnicity, education, parity, smoking, alcohol use, mode of delivery, infant sex, gestational age, and birth weight z score. For birth weight as the outcome, the effect was presented as z score change. The adjusted % change was calculated from the regression coefficient for the dependent variable (y) in log scale per log unit increase in each vitamin measurement (x): because the regression coefficient (β) represents the proportion of change in y in the original scale: log y1 − log y0 = β, then log (y1/y0) = β, thus y1/y0 = e^β^, and the percentage change is (e^β^ −1) * 100%.

^b^There was a significant interaction between infant sex and cord blood retinol in relation to IGF-I (*P* = 0.003): the association was present in girls [28.0 (11.1–47.5), *P* < 0.001)], but not in boys (*P* = 0.75); otherwise, no significant interactions affecting these effect estimates of interest.

*P < 0.05.

There was a significant interaction (p = 0.003) between infant sex and cord blood retinol in relation to IGF-I concentration. Adjusting for maternal and newborn’s characteristics, one log unit increase in cord plasma retinol was associated with a 28.0% (11.0–47.5%) increase in IGF-I in girls (n = 117, *P* < 0.001), but there was no association in boys (n = 131, *P* = 0.75). There were no other significant interactions affecting the associations of retinol, β-Carotene, γ- and α-tocopherols with leptin, adiponectin, IGF-I and IGF-II in newborns.

Similar findings were observed if the analyses were restricted to non-GDM and term pregnancies (n = 215).

## Discussion

### Main Findings

To our knowledge, the present study is the first to observe that there was a sex-specific association between cord plasma retinol and IGF-I, and that cord plasma α-tocopherol was negatively associated with adiponectin, while γ-tocopherol was negatively associated with leptin in neonates. These observations suggest that vitamin E and A nutritional status may matter for metabolic health in early life in humans.

### Data interpretation and comparisons with findings in previous studies

Our and other studies have shown that leptin, but not adiponectin may affect fetal insulin sensitivity^19,23^. Little is known about whether any nutrients may affect fetal leptin and adiponectin levels. In the present study, we observed that cord plasma α-tocopherol was negatively associated with adiponectin, while γ-tocopherol was negatively associated with leptin even after adjusting for potential confounders. The observed negative associations suggest that α-tocopherol might suppress adiponectin expression, while γ-tocopherol might suppress leptin expression in early life. Adiponectin and leptin are important adipokines in regulating insulin sensitivity^24,25^. One might speculate that these early life associations may have long-term implications for metabolic health in later life. Indeed, recently, Simpson *et al*. reported that cord blood leptin was positively associated with fat mass, waist circumference and body mass index at age 9, whereas cord blood adiponectin was positively associated with fat mass and waist circumference at age 17^26^. Thus, our observed negative associations of cord blood vitamin E with leptin and adiponectin suggest a potential beneficial role of vitamin E in early life in preventing the development of childhood obesity. The mechanisms are unknown and deserve further studies.

Our study is the first to report that cord plasma retinol was significantly correlated to IGF-I in girls but not in boys. A recent study in mice suggests that vitamin A deficiency may affect fetal islet development^27^. It has been reported that retinol may have a sex-specific impact on the activity of glycine N-methyltransferase - a key liver protein involved in regulating hepatic S-adenosylmethionine and folate metabolism^28^. Folate is a well-known methyl donor that may affect cell epigenetic changes. Interestingly, treatment of IGF-I deficient mice with growth hormone significantly suppressed glycine-N-methyltransferase activities^29^. One might speculate that retinol may modify early life expression of IGF-I in a sex-specific way through epigenetic changes. However, this interpretation is largely speculative. More studies are required to understand the mechanisms.

Vitamins A and E cannot be synthesized de novo in the fetus^30^, therefore, their fetal/cord blood concentrations are an excellent indicator of maternal/fetal nutritional status. Tocopherols, carotenoid and retinol are stable in plasma sample during long-term storage at −70 °C^31,32^. Most studies on vitamins A and E in pregnancy have been focused on the association with fetal growth, and the findings have been inconsistent^33–35^. Similar to our study, Masters and colleagues reported that cord blood retinol concentrations were positively associated with birth weight^33^, but Weber and colleagues reported that maternal retinol concentrations were inversely associated with birth weight^34^. Masters *et al*. and Scholl *et al*. reported that maternal α-tocopherol concentrations were positively associated with birth weight, but Poston and colleagues found that prenatal supplementation with α-tocopherol and vitamin C was associated with elevated risk of low birth weight^8^. We did not observe any significant association between α- or γ-tocopherol and birth weight. The reasons behind these discrepancies are unclear, and might be partly due to differences in the accuracies of vitamins and birth weight measurements. It is noteworthy that in our study, vitamin A and E measurements were pretty robust (low intra- and inter-assay CVs), and that birth weight was measured by research nurses using a reliable electronic weighing scale. Some previous studies used birth weight data from medical charts which may be less reliable than measured values. Poorer data accuracies might increase random variations and decrease the chances to detect a true association. Alternatively, there might be true population-specific associations in that an association may manifest only a particular population due to specific genetic and environmental contexts.

Taking together, our observations suggest the importance of retinol, α- and γ- tocopherols for metabolic health in early life in humans.

### Limitations

The study is observational in nature. We could not assume that the observed associations are causal. Further intervention studies are required to confirm causality. It should be noted that the normal reference values for vitamins A and E in cord blood are not available, and our observed associations were mostly moderate. Thus, caution is warranted in data interpretation concerning the potential clinical significance of study findings. The study was based on a Canadian birth cohort of largely White subjects. Independent studies in other countries/regions are required to understand the generalizability of the study findings.

## Conclusions

There may be a sex-specific association between retinol and IGF-I, a negative association between α-tocopherol and adiponectin, and a negative association between γ-tocopherol and leptin in early life in humans.

## Electronic supplementary material

STROBE checklist

## References

[1] Becker W (2004). Nordic Nutrition Recommendations 2004-integrating nutrition and physical activity. Scand J Nutr.

[2] Otten, J. J., Hellwig, J. P. & Meyers, L. D. *Dietary reference intakes: the essential guide to nutrient requirements –part III vitamins and mineral*s. National Academies Press, pp 167–462, 2006.

[3] Ford ES, Schleicher RL, Mokdad AH, Ajani UA, Liu S (2006). Distribution of serum concentrations of α-tocopherol and γ-tocopherol in the US population. Am J Clin Nutr.

[4] Tanumihardjo SA (2016). Biomarkers of Nutrition for Development (BOND)-Vitamin A Review. J Nutr.

[5] Gagne A, Wei SQ, Fraser WD, Julien P (2009). Absorption, transport, and bioavailability of vitamin e and its role in pregnant women. Obstet Gynaecol Can.

[6] Tanumihardjo SA (2011). Vitamin A: biomarkers of nutrition for development. Am J Clin Nutr.

[7] Jiang Q, Christen S, Shigenaga MK, Ames BN (2001). γ-Tocopherol, the major form of vitamin E in the US diet, deserves more attention. Am J Clin Nutr.

[8] Poston L, Briley AL, Seed PT, Kelly FJ, Shennan AH (2006). Vitamin C and vitamin E in pregnant women at risk for pre-eclampsia (VIP trial): randomised placebo-controlled trial. Lancet.

[9] Martín-Estal I, de la Garza R, Castilla-Cortázar I (2016). Intrauterine Growth Retardation (IUGR) as a Novel Condition of Insulin-Like Growth Factor-1 (IGF-1) Deficiency. Rev Physiol Biochem Pharmacol.

[10] Diener, A. & Rohrmann, S. Associations of serum carotenoid concentrations and fruit or vegetable consumption with serum insulin-like growth factor (IGF)-1 and IGF binding protein-3 concentrations in the Third National Health and Nutrition Examination Survey (NHANES III). *J Nutr Sci* **5** (2016). 10.1017/jns.2016.1 PMC4791518 27313849

[11] García OP (2013). Zinc, iron and vitamins A, C and E are associated with obesity, inflammation, lipid profile and insulin resistance in Mexican school-aged children. Nutrients.

[12] Martins Gregório B (2016). C. The potential role of antioxidants in metabolic syndrome. Curr Pharm Des.n.

[13] Alcalá M (2015). Vitamin E reduces adipose tissue fibrosis, inflammation, and oxidative stress and improves metabolic profile in obesity. Obesity.

[14] Sugiura M, Nakamura M, Ogawa K, Ikoma Y, Yano M (2015). High-serum carotenoids associated with lower risk for developing type 2 diabetes among Japanese subjects: Mikkabi cohort study. BMJ Open Diabetes Res Care.

[15] Luo Z-C (2012). *Maternal and fetal IGF-I and IGF-II levels*, *fetal gro*wth, and gestational diabetes. The Journal of Clinical Endocrinology & Metabolism.

[16] Tsai PJ (2004). Cord plasma concentrations of adiponectin and leptin in healthy term neonates: positive correlation with birthweight and neonatal adiposity. Clin Endocrinol.

[17] Luo Z-C (2010). Maternal glucose tolerance in pregnancy affects fetal insulin sensitivity. Diabetes care.

[18] Levy E (2000). Altered lipid profile, lipoprotein composition, and oxidant and antioxidant status in pediatric Crohn disease. Am J Clin Nutr.

[19] Luo ZC (2013). Maternal and fetal leptin, adiponectin levels and associations with fetal insulin sensitivity. Obesity.

[20] American Diabetes Association. Diagnosis and classification of diabetes mellitus. *Diabetes care* **32**, S62-S67 (2009). 10.2337/dc09-S062 PMC2613584 19118289

[21] Kramer MS (2001). A new and improved population-based Canadian reference for birth weight for gestational age. Pediatrics.

[22] Austin PC, Steyerberg EW (2015). The number of subjects per variable required in linear regression analyses. J Clin Epidemiol.

[23] Catalano PM, Presley L, Minium J, Hauguel-de Mouzon S (2009). Fetuses of obese mothers develop insulin resistance in utero. Diabetes care.

[24] Rabe K, Lehrke M, Parhofer KG, Broedl UC (2008). Adipokines and insulin resistance. Molecular medicine (Cambridge, Mass.).

[25] Zhang H, Zhang C (2010). Adipose “talks” to distant organs to regulate insulin sensitivity and vascular function. Obesity.

[26] Simpson J (2017). Programming of Adiposity in Childhood and Adolescence: Associations With Birth Weight and Cord Blood Adipokines. J Clin Endocrinol Metab.

[27] Chien CY (2016). Maternal vitamin A deficiency during pregnancy affects vascularized islet development. J Nutr Biochem.

[28] McMullen MH, Rowling MJ, Ozias MK, Schalinske KL (2002). Activation and induction of glycine N-methyltransferase by retinoids are tissue-and gender-specific. Arch Biochem Biophys.

[29] Brown-Borg HM, Rakoczy SG, Uthus EO (2005). Growth hormone alters methionine and glutathione metabolism in Ames dwarf mice. Mech Ageing Dev.

[30] Didenco S (2011). Increased vitamin E intake is associated with higher alpha-tocopherol concentration in the maternal circulation but higher alpha-carboxyethyl hydroxychroman concentration in the fetal circulation. Am J Clin Nutr.

[31] Gross MD, Prouty CB, Jacobs D (1995). Stability of carotenoids and alpha-tocopherol during blood collection and processing procedures. Clin Chem.

[32] Hankinson S (1989). Effect of transport conditions on the stability of biochemical markers in blood. Clin Chem.

[33] Masters ET (2007). Relation between prenatal lipid-soluble micronutrient status, environmental pollutant exposure, and birth outcomes. Am J Clin Nutr.

[34] Weber D (2014). Oxidative stress markers and micronutrients in maternal and cord blood in relation to neonatal outcome. Eur J Clin Nutr.

[35] Scholl TO, Chen X, Sims M, Stein TP (2006). Vitamin E: maternal concentrations are associated with fetal growth. Am J Clin Nutr.

